# Prediction of stability changes upon mutation in an icosahedral capsid

**DOI:** 10.1002/prot.24859

**Published:** 2015-08-01

**Authors:** Samuel J. Hickman, James F. Ross, Emanuele Paci

**Affiliations:** ^1^ Astbury Centre for Structural Molecular BiologyUniversity of LeedsLeedsLS2 9JTUnited Kingdom

**Keywords:** capsids, stability, self‐assembly, modeling, simulation, free energy of association, protein engineering, lumazine synthase

## Abstract

Identifying the contributions to thermodynamic stability of capsids is of fundamental and practical importance. Here we use simulation to assess how mutations affect the stability of lumazine synthase from the hyperthermophile *Aquifex aeolicus*, a *T* = 1 icosahedral capsid; in the simulations the icosahedral symmetry of the capsid is preserved by simulating a single pentamer and imposing crystal symmetry, in effect simulating an infinite cubic lattice of icosahedral capsids. The stability is assessed by estimating the free energy of association using an empirical method previously proposed to identify biological units in crystal structures. We investigate the effect on capsid formation of seven mutations, for which it has been experimentally assessed whether they disrupt capsid formation or not. With one exception, our approach predicts the effect of the mutations on the capsid stability. The method allows the identification of interaction networks, which drive capsid assembly, and highlights the plasticity of the interfaces between subunits in the capsid. Proteins 2015; 83:1733–1741. © 2015 The Authors. Proteins: Structure, Function, and Bioinformatics Published by Wiley Periodicals, Inc

## INTRODUCTION

Many biological processes, such as cell scaffolding, signaling cascades, transcriptional initiation, and the immune response are mediated by the formation of protein−protein interactions. A stable complex corresponds to a conformation (or ensemble thereof) where the total free energy of the system is lowest relative to any other state. Harnessing the principles underlying the formation of stable macromolecular complexes is a required step to design *de novo* complexes for biotechnological applications.

Some remarkable successes in designing symmetric protein cages have been reported recently. The Yeates group has succeeded in designing a “protein cube”^1^ by fusing two protein domains together via a rigid helix. Protein cages have also been recently obtained via computational screening of protein scaffolds based on designing novel protein−protein interactions through Rosetta.^2^ A different approach consists in using coiled coils to facilitate the symmetrical assembly without redesigning protein−protein interfaces.^3^ This approach allows the formation of a protein cage, which is able to change its size through contraction of the subunits via intrinsic flexibility in the regions in which the coiled coils join to the main globular subunits.

A large number of proteins spontaneously self‐assemble into high‐order protein cages with hollow interiors.^4^, ^5^, ^6^ In nature, capsids are most often found as containers for the encapsulation of molecules, of which viruses are the best‐known example. Capsids are also potential vehicles for small molecule delivery,^7^ reaction vessels,^8^ imaging and biosensing.^9^ Like most oligomeric proteins, capsids are held together by noncovalent interactions at the subunit interface and form symmetric assemblies. Any interface formed between two subunits in a symmetric assembly corresponds to one of the operations of the symmetry group. As a result, a small number of conserved residues are responsible for most of the interaction energy^10^ and as such their design is facilitated relative to that of asymmetric assemblies.^11^

Capsids are in general thermodynamically very stable, but also dynamic. One aspect of capsids' highly dynamic nature, capsid breathing, has been observed experimentally^12^ and in simulations^13^ and is related to maturation, function, and possible inhibition mechanisms of viruses. Fluctuations in the capsid state likely contribute to the thermodynamic stability of capsids through a smaller entropic penalty relative to the state in which the capsid components are dissociated.^14^

**Figure 1:**
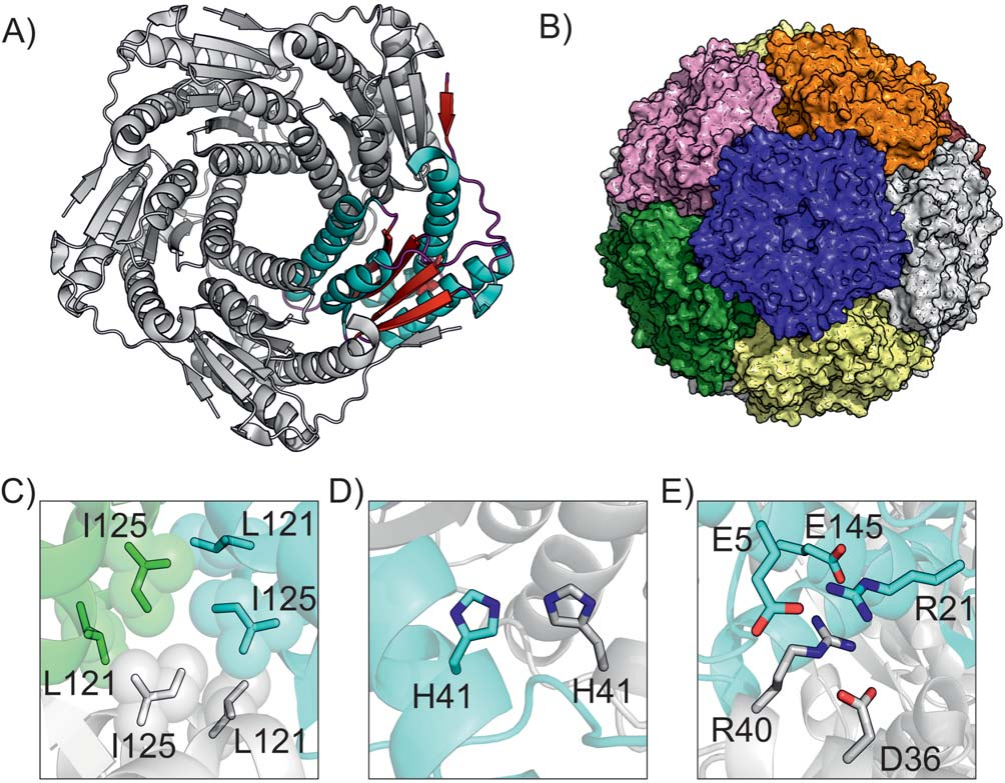
A: Structure of the AaLS pentamer, with an individual monomer highlighted in color. **B**: The icosahedral capsid formed by 12 pentamers. The bottom panel shows the major contact points at the AaLS pentamer interface, (**C**) the hydrophobic cluster, (**D**) a hydrogen bonding site, and (**E**) ionic network. The colors show residues from different pentamers.

Lumazine synthase (LS), an enzyme that catalyzes the penultimate step of riboflavin synthesis,^15^ is an example of a nonviral, naturally empty capsid. Interestingly, LS has been found to form different quaternary structures in different species.^16^ In all species, LS forms a homopentamer subunit. In some organisms these homopentamers can oligomerise to form dimers or dodecamers. In the thermophilic rod‐shaped bacterium, *Aquifex aeolicus,* LS (AaLS) forms an assembly [Fig. 1(B)] of 12 identical rigid homopentamers [Fig. 1(A)] that has been shown to be extraordinarily stable (*T*_m_ = 120 °C).^17^ Historically this assembly was designated as “icosahedral” as both icosahedra and dodecahedra belong to the same point group symmetry.^18^ Due to the high thermostability of AaLS it has been widely used as a system to model encapsulation^19^, ^20^, ^21^, ^22^ and as a model for investigating interfacial interactions in capsids.^16^, ^17^, ^23^

From the structure of AaLS from *Aquifex aeolicus* [PDB: 1HQK, Fig. 1(A)],^24^ three inter‐pentamer interaction regions were identified; a hydrophobic cluster (L121 and I125), a hydrogen bonding site (H41) and an ionic network (E5, R21, D36, R40, and E145) [Fig. 1(C−E)], which were considered to be essential for driving capsid assembly.^23^ Point mutations introduced into these regions (summarized in Table 1) showed that destabilization of the hydrophobic cluster was necessary to disrupt the dodecamer formation completely;^23^ removal of ionic interactions or the hydrogen bonding site alone was insufficient to prevent capsid formation.

**Table 1:** Mutations Experimentally Assessed in Ref 23

Mutant	Ionic network		H‐bond	Hydrophobic cluster		Oligomeric state
WT	R21	R40	H41	L121	I125	Capsid
W2		R40E	H41E			Capsid
W3	R21E	R40E	H41E			capsid
W4			H41R	L121R		pentamer
W5		R40E	H41E	L121E		pentamer
W6		R40E	H41E		I125E	pentamer
W7		R40S	H41S		I125S	pentamer
W8		R40S	H41L	L121E		pentamer

AaLS is an ideal model for studying capsid interfaces by computational approaches for various reasons: the crystal structure has been solved at high resolution (1.75 Å); unlike viruses the capsid is hollow and therefore does not require a contribution from nucleic acid interactions to form and experimental data is available on point mutations that prevent capsid assembly.

Computational studies of virus capsids have played an important role in understanding the stability and dynamics of these systems. Some notable examples include the first all atom simulation of the entire Tobacco Mosaic Virus, with and without a modelled RNA core,^25^ a 1 µs simulation of the whole Satellite Tobacco Necrosis Virus^26^ in the presence and absence of Ca^2+^ and a remarkable 64 million atom model of the mature HIV‐1 capsid.^27^

Although advances in parallel computing has permitted these huge all atom systems to be simulated, the symmetrical nature of icosahedral capsids allows the use of rotational symmetry boundary conditions (RSBC)^28^ in which a single asymmetric unit can be simulated under icosahedral symmetry conditions, effectively representing the entire capsid with negligible interfacial influence of the imposed symmetry image.^29^ To date, this method has been successfully utilised to study the conformational and elastic properties,^29^, ^30^ pH effects,^31^ and antiviral interactions^13^, ^32^ of viral capsids. Molecular modeling approaches to study spherical capsids been discussed in a recent review.^33^

In this study, we test our ability to estimate the effect of mutations on the stability of the AaLS capsid. We chose to simulate the AaLS capsid in a crystal environment by imposing the crystal symmetry on the asymmetric unit containing a single pentamer [Fig. 1(A)]. In this way the whole dodecahedral capsid (1 MDa) can be simulated in explicit solvent, albeit in a crystal environment, by simulating only one asymmetric unit containing a solvated pentamer (Supporting Information Fig. S1).

We show that empirical estimators of free energy of association can provide a good prediction of the experiment when averages over an ensemble are taken. Additionally, simulation is instrumental to assess how mutations affect the structure and dynamics of the AaLS capsid.

## METHODS

### Simulation

Simulations were carried out using CHARMM (version 38) with the all atom CHARMM27 force field in the NPT ensemble at 300 K temperature and 1 atm pressure; the nonbonded cut‐off was set to 12 Å, and Ewald summation was used for electrostatics. Simulations were started from the asymmetric unit of AaLS containing a homopentamer (PDB 1NQU). We applied the symmetry operator for space group I23 to obtain a cubic unit cell with side 180.56 Å containing two dodecamers and cubic periodic boundary conditions (see Supporting Information for details on the simulation set‐up and Supporting Information Figure S1 for a representation of the asymmetric unit and the unit cell). Thus we effectively simulated an infinitely repeated body‐centered cubic crystal with two capsids in each unit cell. Additional water and counter‐ions were added to the asymmetric unit so that the system is neutral and the density identical to that of the crystal. The system was then slowly heated (for 60 ps) and equilibrated (for another 120 ps). The simulation time for the wild type AaLS was 23 ns.

The LS mutations highlighted in Table 1 were applied to the AaLS structure using the “BuildModel” feature of FoldX.^34^ BuildModel selects the energetically optimal rotamer and then iteratively selects optimal rotamers for residues surrounding the mutated residue returning the conformation with lowest Δ*G*_stability_. The AaLS mutants were simulated under the same conditions as the wild‐type protein for 7 − 13 ns depending on the mutant. Simulation of a single fully solvated pentamer was also carried out for 9.4 ns. In all cases, conformations were created for each ps of simulation.

### Estimation of Binding Free Energy

The free energy of capsid formation, or more in general, of association of a complex, is by definition the difference between the free energy of the entire complex minus that of the individual components:

$${\Delta G}_{association}=G_{complex}‐\sum_{component\;i}^{N}G_{i}$$

If Δ*G*_association_ can be estimated for both the mutant and the wild‐type protein their difference

$${\Delta \Delta G}_{association}={\Delta G}_{association(mutant)}‐{\Delta G}_{association(wild‐type)}$$

provides a prediction of how much the mutation (de)stabilizes the capsid state.

Various approaches to estimate the stability of proteins and complexes have been proposed based on empirical force fields that provide an estimation of the free energy difference between two structures representing two states. In this work we chose to estimate free energies of association using PISA.^20^, ^35^ PISA estimates the free energy of dissociation of quaternary structures that appear when crystal symmetry is applied to the asymmetric unit. The approach has been proposed to discriminate between protein−protein interactions resulting from crystal packing and biologically relevant interactions that stabilize the quaternary structure of the protein. These features make it straightforwardly applicable to the case discussed in this article. Snapshots from the trajectory (asymmetric units containing a solvated pentamer) were saved in PDB format to which the CRYST1, ORIGXn, and SCALEn headers were added with the appropriate cell parameters for that time point and passed to PISA for evaluation. PISA^35^ provides an output of possible quaternary structures, one made of five monomers and one made of 12 pentamers (the icosahedral capsid), together with an associate free energy of dissociation. The free energy of capsid formation was also estimated using two alternative empirical estimators, FoldX^34^ and Rosetta.^36^ Results for these compare worse with the experiment than those obtained with PISA and are reported in Supporting Information (Supporting Information Figure S2).

## RESULTS

**Figure 2:**
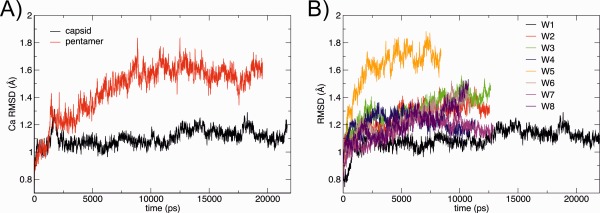
A: RMSD of the Cα atoms in the simulation of the wild‐type AaLS in the crystal environment and as a single pentamer in solution. **B**: RMSD of each mutant simulated in the crystal. [Color figure can be viewed in the online issue, which is available at wileyonlinelibrary.com.]

The AaLS capsid in the crystal state and a solvated pentamer were simulated as described in Methods. Figure 2(A) shows the RMSD of the wild‐type protein relative to the crystal structure in these two different environments. The RMSD shows that when in the capsid, the pentamer drifts very little from the crystal structure (RMSD < 1.2 Å). When simulated as a single pentamer in solution the larger RMSD values are due to rearrangements of residues at the hydrophobic surfaces of the interface between pentamers, and in particular in the highly conserved flexible loop between α‐helix 4 and 5 in capsid forming LS (residues 128 − 134).

The mutants used experimentally to investigate surface interactions (listed in Table 1) were generated using FoldX.^37^ Each mutant was simulated as a capsid for ∼10 ns by implying the crystal lattice of the I23 space group through periodic boundaries. For all the mutants the RMSD increases more than the wild‐type protein [Fig. 2(B)], which suggests local rearrangement around the point mutations.

**Figure 3:**
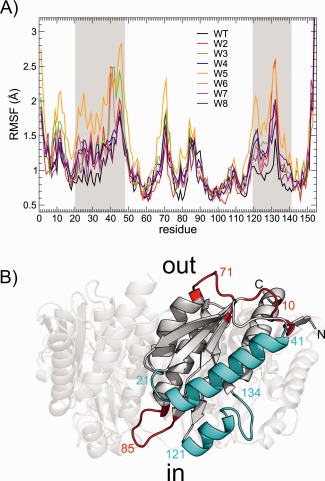
A: Root mean square fluctuations around the average structure. Shaded areas indicate residues involved in pentamer−pentamer interactions. **B**: Secondary structure cartoon representation of a wild‐type AaLS monomer. Regions involved in inter‐pentamer contact are colored in teal, flexible loops not involved in inter‐pentamer contact are colored red. Numbers indicate the residue number within the structure. [Color figure can be viewed in the online issue, which is available at wileyonlinelibrary.com.]

Figure 3(A) shows the root means square fluctuations (RMSF) of the residues within the monomeric subunits of the pentamer. The smallest fluctuations are observed for the wild‐type protein; in particular, RMSF are smaller in correspondence of beta sheets, which likely constitutes to the rigid region of the capsid. For the wild‐type AaLS, apart from loop regions exposed to the solvent [Fig. 3(B)], fluctuations are higher for those residues involved in the contact between pentamers. Mutations increase the local conformational variability, particularly in the regions where mutations occur, that is, at the interface between pentamers.

**Figure 4:**
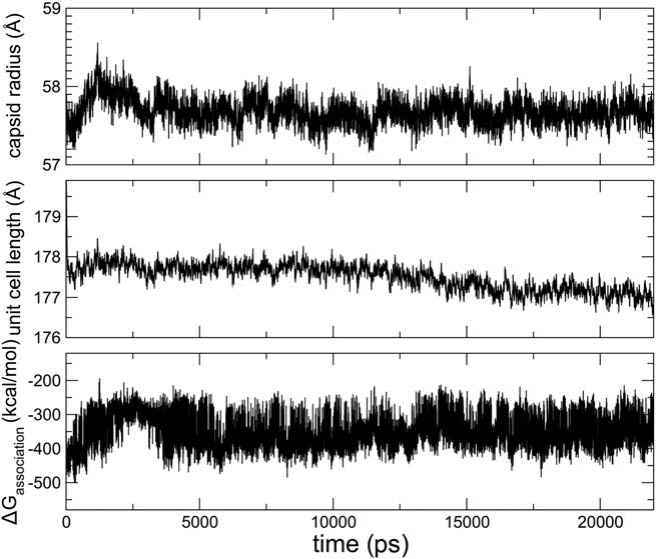
Time series of the estimated Δ***G***_association_ of the capsid radius and of the unit cell length of the crystal. The capsid radius is the radius of an inscribed sphere tangent to each of the dodecahedron's faces and has been calculated using the relation between the distances between the centers of mass of neighboring pentamers. Both the capsid radius and the unit cell length slightly decrease during the equilibration from the crystal structure values of 59.9 Å and 180.57 Å, respectively. From the fluctuations of capsid radius and unit cell length an estimation of the isothermal compressibility can be obtained using the relation $\chi_{T}=〈{\delta V}^{2}〉{\left(k_{B}T〈V〉\right)}^{‐1}$ 41; the compressibility of the capsid turns out to be 0.3 GPa^−1^, close to the estimate for the intrinsic compressibility of a globular protein (∼0.2 GPa^−1^ compared to 0.5 GPa^−1^ for liquid water that is less compressible than organic liquids and polymer melts).^42^, ^43^, ^44^

Once the RMSD reached a plateau [Fig. 2(B)], structures were collected every 1 ps. Structures and the associated crystal parameters were entered, in PDB format, to PISA,^35^ that identified complexes resulting from the application of the crystal symmetry and estimated a free energy of dissociation. For each of the frames analyzed, PISA identifies a stable structure constituted by 12 pentamers. The “time series” of Δ*G*_association_ for the wild‐type, together with the time series of the capsid radius and the unit cell length is presented in Figure 4. The Δ*G*_association_ estimated for different frames of the trajectory can vary by ±200 kcal/mol. This is unexpected given that conformations of the pentamers vary marginally (the RMSD between pairs of conformations is always lower than 1.3 Å). The breathing of the capsid, reflected in the fluctuations of the capsid radius also shown in Figure 4, may in part explain the fluctuations of the estimated Δ*G*_association._

**Figure 5:**
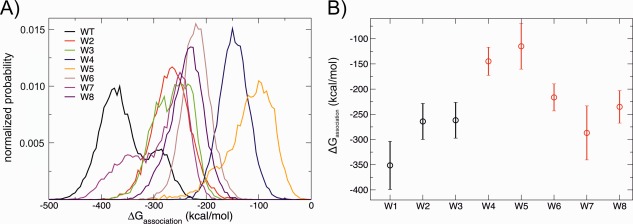
A: Distribution of values of Δ*G*_association_ estimated using PISA^35^ for each snapshot of the simulation; results shown for the wild‐type AaLS and the seven mutants listed in Table 1. **B**: The average of the estimated values of Δ*G*_association_ for each variant (error bars correspond to one standard deviation) Data points shown in red correspond to mutants that experimentally disrupted capsid formation. [Color figure can be viewed in the online issue, which is available at wileyonlinelibrary.com.]

From the time series of Δ*G*_association_ we determined the distribution of Δ*G*_association_ [Fig. 5(A)]. Distributions were remarkably broad, consequence of the fact that Δ*G*_association_ estimated for different frames of the trajectory can vary considerably due to the fluctuations of interface residues. As expected, the distribution of Δ*G*_association_ for the wild‐type AaLS has the lowest modal value of all.

In Figure 5(B) the average Δ*G*_association_ is shown for the wild‐type AaLS and seven mutants. The wild‐type protein forms the most stable capsid of all, with an average Δ*G*_association_ of −350 kcal/mol (close to the PISA estimation for the crystal structure, −395 kcal/mol), which demonstrates the remarkably high stability one of the most thermostable capsids known to date.^23^

**Figure 6:**
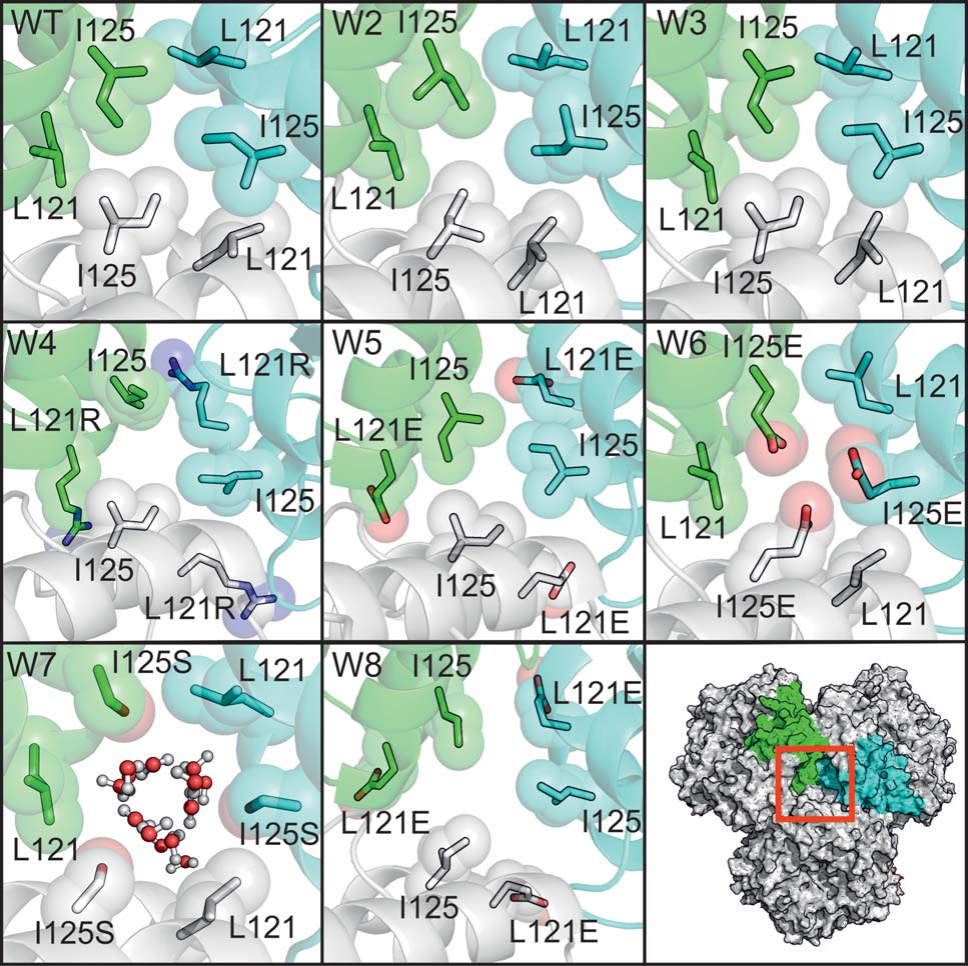
Hydrophobic cluster of AaLS variants after 10 ns of simulation. Mutated residues are labeled. For W7 the waters occupying the cavity are shown as ball and stick. [Color figure can be viewed in the online issue, which is available at wileyonlinelibrary.com.]

For mutants W2 and W3, the capsid assembly was less stable than for the wild‐type AaLS, but only marginally (about 80 kcal/mol); both of these mutants were experimentally found to form capsids despite the disruption of both the hydrogen bonding site and ionic network. Thus, not only the loss of a single arginine (W2), but also the complete removal of both arginines (R21 and R40) (W3) from the ionic network [Fig. 1(E)] does not dramatically destabilize the capsid. The simulation provides an explanation of this: in W3, R52 (which is not involved in the ionic network in the wild‐type protein or mutant W2) repositions its side chain to the glutamate‐rich region, forming inter‐pentamer polar contacts (Supporting Information Figure S3). In both mutants no residues in the hydrophobic cluster were mutated; indeed, during the duration of the simulations the hydrophobic cluster remains tightly packed as in the wild‐type AaLS (see first three panels of Fig. 6).

Mutants W4, W5, W6, W7, and W8 have additional polar mutations in the hydrophobic cluster and have been found experimentally to be in pentameric form in solution, that is, do not form capsids. For mutants W4 and W5 we estimate a Δ*G*_association_ for the capsid state that is more than 200 kcal/mol less favorable than for the wild‐type protein, in accordance with the experimental result.

Mutants W6 and W8 were also destabilized by the mutation in the hydrophobic cluster, but the comparison with the experiment is less clear‐cut as the estimated Δ*G*_association_ is only slightly more unfavorable than those of mutants W2 and W3, for which the capsid is the stable state (∼120 kcal/mol less favourable than the wild‐type protein).

Based on the estimated Δ*G*_association_, mutant W7 is predicted to be in a capsid state, in disagreement with the experimental observation. Possible reasons of this disagreement are discussed below.

The structural changes in the hydrophobic cluster upon mutation are summarized in Figure 6. In variants W4, W5, and W8 the leucine at position 121 is mutated to a polar residue. Because residue 121 occurs within a loop, the polar side chains have more freedom to reposition away from the hydrophobic core, which eliminates the tight packing seen in the wild‐type AaLS. In mutants W6 and W7, the isoleucine at position 125 is mutated to a polar residue. Because of the position in the middle of the alpha helix, residues at this position have less freedom to reposition their side‐chains which is evident by the smaller RMSF value compared to the 121 position [Fig. 3(A)]; as a consequence in W6 the glutamates have the carboxylate groups in proximity of each other.

In W7, the smaller excluded volume of serines results in a cavity in the hydrophobic pocket that is continuously occupied by 5 to 6 waters throughout the simulation (Fig. 6). W7 is the only variant in which the disrupted hydrophobic cluster is hydrated; in mutants W4, W5, and W8 residue I125 occludes the cavity to the surrounding solvent. The presence of a hydrated hydrophobic pocket may be an artifact of the simulation and this may explain why the capsid state of W7 is predicted to be stable in contrast with the experimental observation. Another possible explanation is that the empirical methods used to evaluate the stability of the capsid state underestimate the destabilizing effect of a hydrated cavity at the interface between pentamers. Interestingly, the free energy of capsid formation estimated using two alternative empirical methods, FoldX and Rosetta, also predicts that the capsid conformation of W7 is more stable than for the mutants W2 and W3 (Supporting Information Figure S2).

## CONCLUDING DISCUSSION

The results presented above show how the stability of the icosahedral capsid of lumazine synthase from *Aquifex aeolicus* is affected by a number of mutations that modify ionic (R21 and R40), hydrogen bonding (H41), and hydrophobic (L121 and I125) interaction sites along the interfaces between the 12 pentamers that constitute the capsid. It was previously found that a mutation in the hydrophobic cluster was necessary to destabilize the dodecahedral capsid form.^23^ Simulations of each of the mutants, where the icosahedral symmetry is imposed to an asymmetric unit containing a single pentamer, show that the ionic network was never completely lost after removal of the arginine residues and flexibility at the pentamer interface allows residues to freely reposition into favorable conformations. Introduction of polar residues into the hydrophobic cluster always leads to disruption of the tight packing of the side chains, and despite the flexibility, the surrounding residues are unable to rearrange into a favorable conformation, leading to the formation of a small cavity; this may explain why the experimental mutations within the hydrophobic cluster were detrimental to capsid formation in AaLS.

Our simulations show that the structure of the wild‐type pentamer deviates very little from the crystal structure throughout the simulation, which is in agreement that capsids contain rigid domains^38^,^39^ and positional fluctuations are largest at the pentamer‐pentamer interface. Capsid breathing is observed, with the radius of the capsid fluctuating by about 2% around the average value. Also in the case of all the mutants deviation from the wild‐type crystal structure is small, but regions directly affected by the mutations fluctuate much more than in the wild‐type structure. Indeed, our approach consisting of simulating a single pentamer and using symmetry to enforce the capsid environment does not allow asymmetric modes of fluctuations; simulation of the entire solvated capsid would be required to observe conformational changes that would break the capsid's symmetry.

To predict the change in free energy upon association of pentamers we used an empirical method that estimates free energy differences in terms of interatomic interactions, and approximations to account for the interaction with the solvent and the various entropic contributions. The values obtained depend remarkably on the conformation; despite the sample of conformations for each wild‐type pentamer differing less than 1.3 Å RMSD, and the radius of the capsid varying less than 2% along the trajectory, the estimated free energy of association varies between −215 and −475 kcal/mol for individual structures on the trajectory.

**Figure 7:**
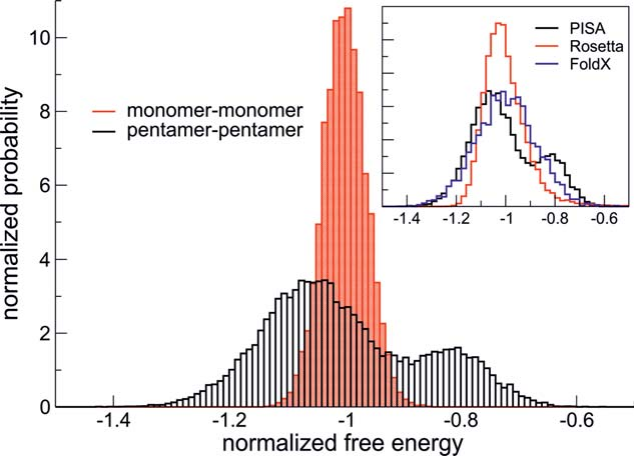
Distribution of values of Δ***G***_association_ estimated using PISA^35^ for each snapshot of the simulation, divided by the average Δ***G***_association_ over the simulation; results shown for the wild‐type AaLS only. In black is shown the result for pentamer−pentamer association (i.e., the free energy gained when 12 pentamers associate to form a capsid), in red the result for monomer−monomer association within one pentamer (i.e., the free energy gained when 5 monomers associate to form a pentamer). In the inset the distribution of values of Δ***G***_association_ estimated using FoldX^34^ and Rosetta^36^ is also shown. [Color figure can be viewed in the online issue, which is available at wileyonlinelibrary.com.]

This is not the case for interactions between monomers in the pentamer. The distribution of estimated free energies (divided by their average) for pentamer−pentamer and monomer−monomer interactions are shown in Figure 7. For pentamer−pentamer interactions this distribution is broad as observed above; for monomer−monomer interactions it is symmetric and considerably narrower. This fact is noteworthy and highlights the dynamic nature of the interface residues between the capsid‐forming units, that is, the pentamers. The highly dynamic interfaces between the pentamers forming the capsid are likely responsible for the resilience of the capsid to potentially disruptive mutations at the interface, but also highlights the difficulty of assessing the stability of a capsid from an energy (or free energy) calculation based on a single structure, either experimentally determined or rationally designed.

Another point worth discussing is the apparent bimodality of the distribution of Δ*G*_association_. The time series of the estimated Δ*G*_association_ (Fig. 4) showed rapid fluctuations around the two values that suggest the presence of two conformational substates. However, the variation in estimated Δ*G*_association_ does not correlate with the variation of global conformational properties such as the RMSD or the capsid radius (also shown in Fig. 4). Local properties that may affect the estimation of Δ*G*_association_ such as the number of hydrogen bonds, salt bridges, and van der Waals contact in the pentamer−pentamer interfaces, also do not confirm the existence of two distinguishable states. One possibility is that the bimodality of the distribution of Δ*G*_association_ depends on the estimation of buried or solvent exposed surface area, that is, small changes in the atomic position may cause a large changes in the surface estimation; and this may reflect a property of the system or it could be an artifact; in all cases we do not suggest that PISA or any other method to estimate Δ*G*_association_ from the coordinates of a complex is exact; we show, however, that taking averages over conformations representing the capsid state is necessary and sufficient to predict the complex effect of mutations on the stability of the complex.

In the inset in Figure 7 are compared the distributions of Δ*G*_association_ estimated using the three different empirical methods, namely PISA, FoldX, and Rosetta. While the bimodality of the distribution is only evident for the PISA estimation, the width of the distributions is similar for the different methods suggesting that its broadness is a robust feature of the system.

By calculating ensemble averages and comparing the experimental mutants with the wild‐type protein, we obtain agreement with the experiment, in the sense that the two mutants that remain in a capsid state have a free energy of association that is closer to the wild‐type than the mutants that do not form capsids. For one single mutant we obtain a clear disagreement. The mutant in question has a serine replacing an isoleucine in the hydrophobic cluster. There are two possible reasons for this disagreement with the experiment; one is that the presence of water in the hydrophobic pocket disrupted by the serine mutation is an artifact of the simulation; another is that all the three different empirical methods used here to estimate free energy of association underestimate how unfavorable the presence of an hydrated cavity in an hydrophobic pocket is.

Prediction of the free energy change upon mutation at the interface between proteins in a complex is important if we want to be able to design novel stable complexes. The results presented here show that the effect of mutations on the capsid‐forming propensity of protein interfaces can be rationally predicted by accounting for the conformation variations at the interface using ensembles generated from molecular dynamics. The approach has several potential applications, such as design of icosahedral capsid out of noncapsid forming pentameric proteins (Ross *et al.,* manuscript in preparation) or rationally stabilizing empty capsids in the development of safe and effective vaccines.^40^

## Supporting information

Supporting Information
