# Supplementary material for: Prediction of stability changes upon mutation in an icosahedral capsid
Source: Proteins. 2015 Aug 1;83(9):1733–41. doi: 10.1002/prot.24859 (PMC4737204; doi:10.1002/prot.24859)
Supplement: Supplementary file 1 — Supporting Information [file PROT-83-1733-s001.docx]

**Supplementary Information for “Interface stability changes upon mutation in an icosahedral capsid” by Samuel J Hickman, James Ross & Emanuele Paci**

**Explicit solvent simulation of AaLS capsid from a single pentamer**

To simulate the entire capsid of AaLS in explicit solvent, we exploit the fact that the biological assembly is an icosahedral capsid generated from the pentamer in the asymmetric unit by the I23 crystallographic symmetry operations. The fully solvated asymmetric unit is shown in Figure S1, A. The IMAGE function of CHARMM allows the use of generic crystal symmetry and together with periodic boundary conditions to simulate an “infinite crystal” in which the cubic unit cell contains two capsids (Figure S1, B). The CHARMM input (and the coordinates of the asymmetric unit containing a pentamer and the solvent) was obtained using the input builder CHARMM-GUI (1) available at <http://www.charmm-gui.org>, entering the following opt: PDB code for structure of the LS pentamer (1NQU); in “symmetry operation options” chose: Generation of Crystal Packing: Space Group I23 (required for asymmetric unit solvation); in “waterbox size option” chose: Asymmetric Unit Solvation (adding 30 neutralizing potassium ions).

The approach we used here is related to the rotational symmetry boundary condition (RSBC) (2) previously used to simulate capsids, and shares with it some advantages and some inconveniences. The main advantage is computational efficiency. One inconvenience is the imposed symmetry and the violation of minimum image convention, which is violated to a different degree by different atoms depending on their position in the asymmetric unit.

Figure S1. A) Solvated asymmetric unit containing one LS pentamer, five phosphate ions, 3272 TIP3P water molecules and 30 potassium ions to guarantee neutrality, for a total of 23961 atoms. B) The unit cell of the AaLS crystal obtained by applying the dodecahedral symmetry to the solvated pentamer (solvent removed for clarity).

In RSBC, the solvent should be treated implicitly, i.e., as a continuum (3, 4). Alternatively, solvent has been treated explicitly and kept inside the boundary with an external potential (5, 6).

## Estimation of free energy differences from structures with alternative methods

Besides PISA (7), two other empirical, previously proposed, methods to estimate the free energy of association from an ensemble of structures were tested. The ensemble of configurations generated from simulations of the wild type and mutants were used to estimate the ∆G_association_, using FoldX (8) and Rosetta (9).

With FoldX the interaction was analysed by the ‘Analyse Complex’ function, while with Rosetta the ‘Interface Analyzer’ function was used. Both these interface analysis tools perform essentially the same task, which is to calculate the ∆G_association_ between a single pentamer and a capsid minus a pentamer. Rosetta offers the option to use a packing algorithm, which repositions sidechains to locations more energetically favourable under the Rosetta energy function. This gives two Rosetta flavours, one where the side-chains are “repacked” (Rosetta-P) and one in which they are not (Rosetta-N).

Figure S1 presents the average ΔG_association_ calculated for each mutant ensemble by each of the four methods. For comparison, the data sets for each method was divided by ∆G_association_ calculated for the wild type.

As discussed in the main text, the estimation obtained through PISA is in agreement with experimental observations with the exception of W7, which is predicted to have a greater stability in the capsid state than W2 and W3, which are observed in the capsid state experimentally.

The estimation obtained with FoldX compares worse with the experiment: the capsid form of W3 is estimated to be unstable.

The estimation obtained with Rosetta-N also compares with the experiment worse than that obtained with PISA: the capsid form of W4 is estimated to be stable.

When the position of the sidechains is optimised, or “repacked”, however, Rosetta provides an estimate of the stability of the capsid state of each mutants that is in agreement with PISA, i.e., apart mutant W7, in agreement with the experiment as far as the ranking on the stability is concerned. The repacking option is in principle not necessary given that the structures for which the free energy change is calculated are “exact” i.e., representative of the ensemble of structures populated at room temperature and not artificially designed, e.g., by computationally docking the pentamers.


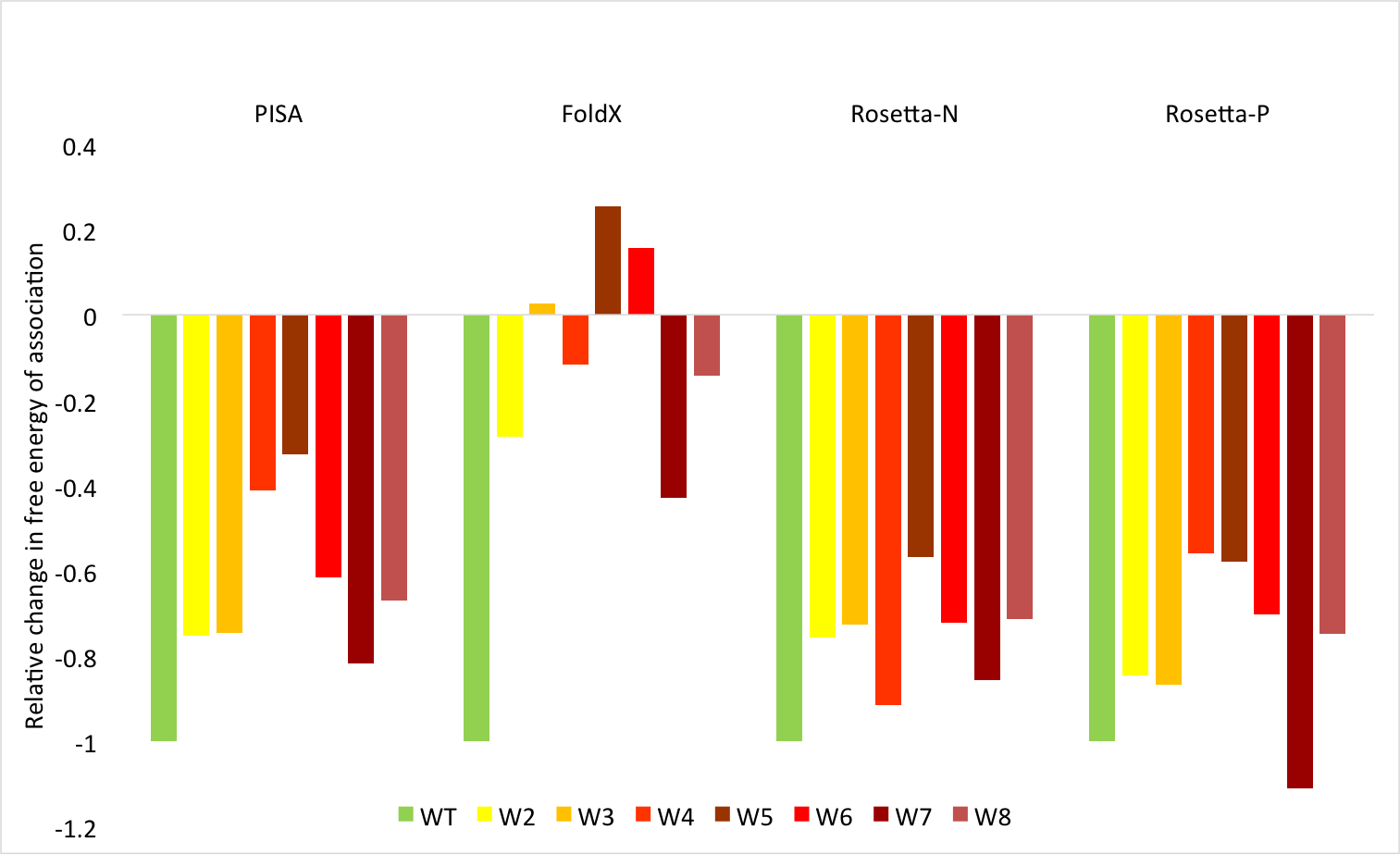


Figure S2. Relative change in free energy of association (i.e., ∆G_association_ of each mutant normalised to the wild-type estimate obtained using different empirical methods: PISA, FoldX and Rosetta.

## How the ionic network repairs itself

After 10 ns simulation of the W3 mutant of AaLS (R21E/R40E/H41E), the average position of the residues within the ionic network was visualised using the software PyMol. It was found that at all of the pentamer interfaces, the position of R52, which in wild-type AaLS has no contact with residues of the ionic network (Figure S3), had its side chain repositioned to become at the centre of the glutamate rich mutant region, forming multiple polar contacts.


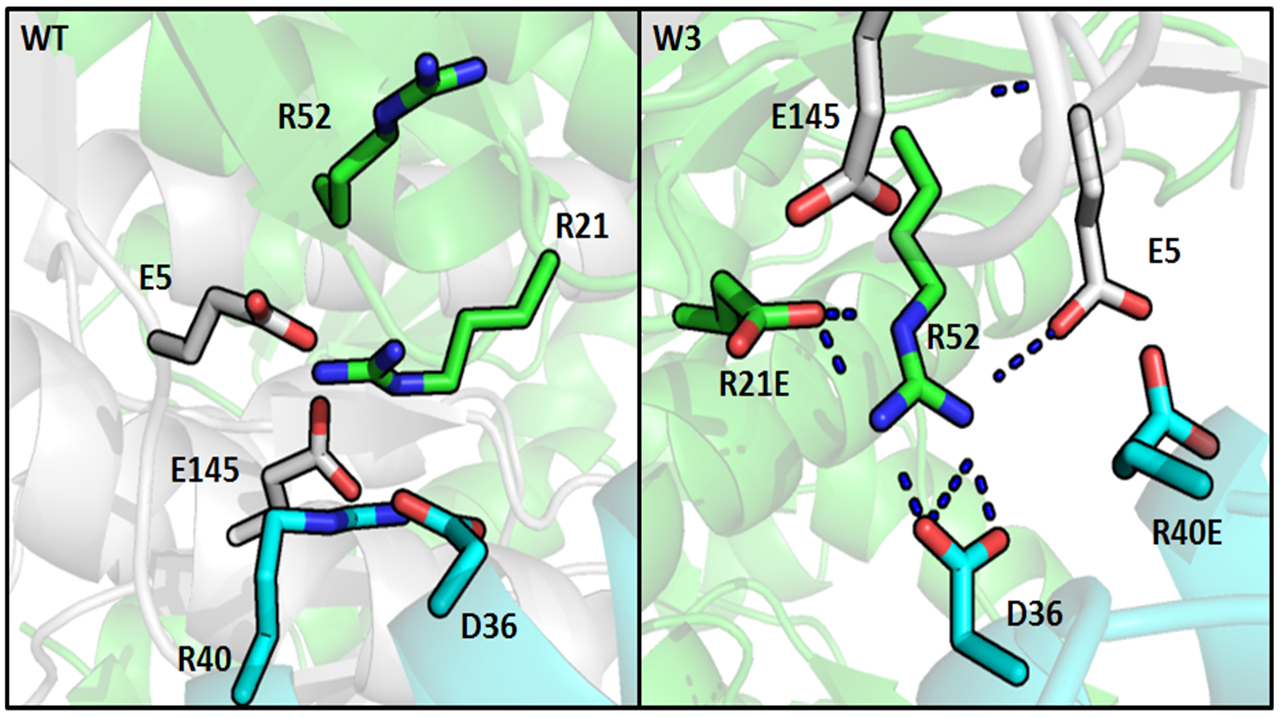


Figure S2 – The position of the side chain of R52 after 10 ns simulation in the wild-type AaLS and the W3 variant (both arginine’s removed from the ionic network). The polar contacts of R52 with surrounding atoms are shown by the blue dashes.

1. Jo S, Kim T, Iyer VG, & Im W (2008) CHARMM-GUI: a web-based graphical user interface for CHARMM. *Journal of computational chemistry* 29(11):1859-1865.

2. Çağin T, Holder M, & Pettitt BM (1991) A method for modeling icosahedral virions: Rotational symmetry boundary conditions. *Journal of computational chemistry* 12(5):627-634.

3. May ER, Aggarwal A, Klug WS, & Brooks CL, 3rd (2011) Viral capsid equilibrium dynamics reveals nonuniform elastic properties. *Biophysical journal* 100(11):L59-61.

4. May ER, Arora K, & Brooks CL, 3rd (2014) pH-induced stability switching of the bacteriophage HK97 maturation pathway. *Journal of the American Chemical Society* 136(8):3097-3107.

5. Roy A & Post CB (2011) Microscopic Symmetry Imposed by Rotational Symmetry Boundary Conditions in Molecular Dynamics Simulation. *J Chem Theory Comput* 7(10):3346-3353.

6. Speelman B, Brooks BR, & Post CB (2001) Molecular dynamics simulations of human rhinovirus and an antiviral compound. *Biophysical journal* 80(1):121-129.

7. Krissinel E & Henrick K (2007) Inference of macromolecular assemblies from crystalline state. *Journal of molecular biology* 372(3):774-797.

8. Schymkowitz J*, et al.* (2005) The FoldX web server: an online force field. *Nucleic acids research* 33(Web Server issue):W382-388.

9. Kaufmann KW, Lemmon GH, Deluca SL, Sheehan JH, & Meiler J (2010) Practically useful: what the Rosetta protein modeling suite can do for you. *Biochemistry* 49(14):2987-2998.
